# Lacosamide as the first add-on therapy in adult patients with focal epilepsy: A multicenter real-world study

**DOI:** 10.3389/fneur.2023.1136814

**Published:** 2023-04-06

**Authors:** Wenyu Liu, Wenjing Li, Peiyu Wang, Hesheng Zhang, Enhui Zhang, Xintong Wu, Dong Zhou

**Affiliations:** ^1^Department of Neurology, West China Hospital, Sichuan University, Chengdu, China; ^2^West China Hospital, Sichuan University, Chengdu, China

**Keywords:** focal epilepsy, lacosamide, adult, efficacy, add-on therapy

## Abstract

**Background:**

Prospective observations on the effectiveness, safety, tolerance, and influence of comorbidity of add-on lacosamide (LCM) therapy are still lacking, especially for domestic generic LCM in China.

**Objective:**

In this multicenter real-world study, we aimed to evaluate lacosamide (LCM) as the first add-on therapy in adult Chinese patients with focal epilepsy that had initially been treated with monotherapy.

**Methods:**

A cohort of consecutive focal epilepsy patients aged over 16 years were enrolled and followed at the multi-epilepsy centers in China. LCM was prescribed as the first add-on therapy. The main outcome measures included seizure frequency and response rate. Data on seizure-free rate, retention rate, scales of depression and anxiety, and adverse events were also collected as additional outcome measures.

**Results:**

A total number of 107 adult subjects (60 men, 56.07%) were enrolled. The mean age was 37.16 ± 15.01 years, and the mean age at seizure onset was 312.35 ± 199.97 months. After the LCM add-on therapy, the ≥50% response rates were 76.19, 81.73, 94.12, and 95.79% at the visit at 4 weeks (visit 2), 8 weeks (visit 3), 16 weeks (visit 4), and 24 weeks (visit 5), respectively, compared to the baseline (visit 1). A total of 34 patients (31.78%) had no seizures during the whole follow-up period. The posttreatment emotional performance of the 97 subjects, defined as generalized anxiety disorder (GAD) and Neurological Disorders Depression Inventory (NDDI) scores, was significantly better than the baseline one. Only one patient suffered from mild dizziness.

**Conclusion:**

LCM as the first add-on therapy in adult focal epilepsy in China was effective and safe. Further prospective studies with long-term follow-up periods are needed to confirm our present findings.

**Clinical trial registration:**

http://www.chictr.org.cn, ChiCTR2100042485.

## Key points

1. After the LCM add-on therapy, the ≥50% response rates were 76.19, 81.73, 94.12, and 95.79% at the visit at 4 weeks (visit 2), 8 weeks (visit 3), 16 weeks (visit 4), and 24 weeks (visit 5), respectively, compared to the baseline (visit 1).
2. A total of 34 patients (31.78%) had no seizures during the whole follow-up period. The retention rates from visits 2–5 were 98.13, 97.19, 95.33, and 88.79%, respectively.
3. The posttreatment emotional performance of the 97 subjects, defined as generalized anxiety disorder (GAD) and Neurological Disorders Depression Inventory (NDDI) scores, was significantly better than the baseline one. Only one patient suffered from mild dizziness.

## Introduction

Epilepsy is one of the most common neurologic disorders, defined by two or more unprovoked seizures, which affects people of all ages. Despite the currently applied therapy with the available appropriate anti-seizure medication (ASM), approximately one-third of the patients with epilepsy continue to suffer from seizure attacks (1, 2), unable to achieve remission from seizure attacks. Therefore, complete and permanent cure application is very difficult to achieve through antiepileptic therapy, which also has adverse side effects (3, 4).

The first proper selection of the antiepileptic drug, based on the specific epilepsy type and at doses >50%, defined as the daily dose, has failed to effectively control seizures, and thus the alteration of regimen may have to be prescribed (5). The alternative options include additional add-on therapy (combination treatment), increased dose of the first drug (another monotherapy), or changing of the first drug (alternative treatment) to obtain seizure control and even achieve a seizure-free outcome (6). A previous study by our research team confirmed that combination therapy may increase the probability of seizure freedom in patients with epilepsy (7) when the first antiepileptic drug failure occurred due to the lack of efficacy. Nevertheless, the application of combination therapy may lead to direct or indirect adverse effects associated with drug metabolism and drug–drug interactions. Therefore, choosing the most suitable add-on ASM with higher effectiveness and safety as the first add-on therapy is of critical importance as it can increase the seizure control rate.

The efficacy of most new-generation ASMs administered as add-on therapy in generalized and focal epilepsy and epileptic syndrome has been confirmed by a number of randomized double-blinded controlled trials and open-label trials (8). Lacosamide (LCM) is one of the new-generation ASMs (9–11) reported to be effective in patients with focal seizures with or without secondary generalization with a maximum licensed dose of 400 mg/day, which can be administered twice daily (12, 13). LCM is rapidly absorbed from the gut and acts on slow inactivation of the sodium channels (14). It has low drug–drug interaction and does not affect the plasma concentrations of other common anti-seizure medications (15). The most commonly observed adverse events (AEs) are dizziness, headache, diplopia, and nausea (16, 17).

However, prospective observations on the effectiveness, safety, tolerance, and influence of comorbidity of add-on LCM therapy are still lacking, especially for domestic generic LCM in China. There is a huge treatment gap in China mainly driven by deficiencies in the delivery of healthcare and also social discrimination. More than one-third of Chinese patients with epilepsy did not receive adequate or appropriate therapy. Furthermore, LCM has not been systematically evaluated in Chinese patients with focal epilepsy. Therefore, in this study, we report our experience with LCM as the first add-on treatment in a cohort of 107 adult patients. The purpose of this study was to evaluate the effect of LCM on seizure control and the quality of life in patients with focal epilepsy.

## Methods

This study was approved by the Ethics Committee of Sichuan University, West China Hospital, Ethics Approval No.: 2020 Annual Review (1199). Patients were enrolled from 14 epilepsy centers in China. The local ethics committee of each epilepsy center or university approved the study; the enrolled patients signed informed consent forms. A total number of 107 adult subjects were enrolled [mean age ± standard deviation (SD): 37.16 ± 15.01 years, 60 men].

The following inclusion criteria were applied: adult patients (age > 16 years) with focal epilepsy; with or without focal to bilateral tonic–clonic seizures; patients who have been taking one antiepileptic drug stably in the past 4 weeks; and at least four episodes every 28 days during the 8-week retrospective baseline period. The recommended initial dose was 50 mg twice daily, which had to be increased based on the specific clinical response and tolerability. The titration scheme consisted of introducing a weekly dose of 50 mg LCM up to the dose of 200–400 mg/day. The exclusion criteria implemented were as follows: LCM has been used in the past; pregnant and lactating women; women of childbearing age who refused contraception during the trial; patients with allergies or allergies to LCM or to any ingredients of excipients; history of status epilepticus within the last 12 months; history of drug/alcohol abuse; history of suicide attempts or suicidal ideation in the past 6 months; current use of antidepressants, anxiolytics, or antipsychotics; a progressive disease that affects the patient's brain and its function; psychogenic non-epileptic seizures; and patients with severe lung and blood system diseases, malignant tumors, low immune function, and primary mental illness.

Information on the following clinical characteristics was collected using standardized questionnaires: demographic data, including age, sex, handedness, and history. The concomitant ASM to which LCM was added was recorded as well as the daily dosage used at the baseline and at each visit during the study period. The main outcome measures related to seizure control included seizure frequency at the baseline and at the different follow-up visits after the LCM add-on therapy administration (reduced seizure attacks during the maintenance period on average of 4 weeks compared to the retrospective baseline period), ≥50% responder rates (percentage of subjects with a ≥50% reduction in seizure frequency within an average 4-week maintenance period compared to the retrospective baseline). The seizure-free rates at the end of each follow-up visit and the retention rate were included as secondary indexes. A safety analysis was performed to establish the incidence and a quantitative criterion and a grading system for adverse drug effects. Anxiety was assessed by the Generalized Anxiety Disorder-7 (GAD-7) scale, and depression was assessed by the Neurological Disorders Depression Inventory NDDI scores. Severe side effects leading to discontinuation were also documented.

This is a post-marketing real-world study, and the inclusion/exclusion criteria are not as strict as those of the registered study. The baseline EKG was not required. However, at the inclusion stage, the doctors/researcher asked the information on EKG and the history of cardiac disease during patient visits. Information on past history was collected using standardized questionnaires including cardiac disease history. In addition, “II-III degree atrioventricular block” is the contraindication of lacosamide. Those patients with abnormal EKG and cardiac disease were not enrolled to participate in this study.

During the follow-up period, 12 adult subjects dropped out, seven of which (58.33%) were lost to follow-up; three (25%) subjects were withdrawn from the trial by the investigator; and two (16.67%) subjects exited the experiment. In total, 13 dropouts occurred at the baseline that would have been to be assessed by both GAD and NDDI.

### Statistical analysis

Descriptive continuous data (age, age onset, height, weight, seizure frequency, drug dosage, and the total scores of the GAD and NDDI outcomes) are expressed as mean ± SD. The pre- and posttreatment differences were compared using the paired *t*-test. Categorical variables are represented as frequency and percentage (%) and were analyzed using the χ2 test or Fisher's exact test. All statistical analyses were performed using SPSS 26.0. A *P*-value of <0.05 was considered to be statistically significant.

## Results

### Basic characteristics

The demographical characteristics of the participants are summarized in Table 1. Of the 107 adult subjects, 60 (56.07%) were men, 107 (100%) were of Han nationality, the mean age at enrollment was 37.16 ± 15.01 years, the mean age at the seizure onset was 312.35 ± 199.97 months, the average height was 167.38 ± 8.14 cm, and the average weight was 64.76 ± 11.33 kg.

**Table 1:** Demographics and clinical characteristics of subjects.

**Characteristics**	***N* = 107**
Age (years)	37.16 ± 15.01^a^
Sex (male/female)	60/47
Age at onset (months)	312.35 ± 199.97^a^
Height (cm)	167.38 ± 8.14^a^
Weight (kg)	64.76 ± 11.33^a^
Family history (±)	0
Drug allergy history	5
Seizure type (focal-only/BTCS)	65/42
**Types of initial ASM**	
Valproate sodium	45
Levetiracetam	26
Oxcarbazepine	18
Carbamazepine	8
Lamotrigine	4
Topiramate	2
Perampanel	1
Valproate magnesium	1
Phenytoin	1
Zonisamide	1

a Shown as mean ± standard deviation (SD).

BTCS, focal to bilateral tonic-clonic seizures.

According to the International League Against Epilepsy (ILAE) 2017 classification and terminology (18), there were 65 cases of only focal seizures and 42 cases of focal to bilateral tonic–clonic seizures. Five subjects had a history of drug allergy, including one (20%) to oxcarbazepine, two (40%) to carbamazepine, one (20%) to lamotrigine, and one (20%) to penicillin. Drug allergy describes clinical adverse reactions manifested as a rash. Eight subjects had a history of comorbidities, including three (37.5%) with anxiety, two (25%) with anxiety and depression, two (25%) with hypertension, and one (12.5%) with meningioma.

Two subjects had abnormal head and neck examination results, including one subject with neck stiffness and one case with bilateral frontal bone loss; one subject was with abnormal spine and limb examination results (post-burn deformity of the right hand); and one subject had abnormal neurological examination results (the muscle tone of the right body was increased). There was no abnormality in the vital signs.

### Combination therapy

A total of 107 (100%) subjects received LCM in combination with another single ASM, in 45 (42.06%), it was combined with sodium valproate; in 26 (24.30%), with levetiracetam; in 18 (16.82%), with oxcarbazepine; in 8 (7.48%), with carbamazepine; in 4 (3.74%), with lamotrigine; in 2 (1.87 %), with topiramate; in 1 (0.93%), with perampanel; in 1 (0.93%), with valproate magnesium; in 1 (0.93%), with phenytoin; and in 1 (0.93%), with zonisamide.

### Main outcome measures

#### Seizure frequency

A descriptive analysis was performed of the seizure frequency of each of the subjects at each visit point. The mean seizure frequency in the past 8 weeks was 12.28 ± 22.47 times/4 weeks, and the mean seizure frequencies of each of the visits from visits 2–5 were 5.13 ± 13.88 times/4 weeks, 3.86 ± 12.83 times/4 weeks, 2.50 ± 12.61 times/4 weeks, and 1.65 ± 10.29 times/4 weeks, respectively. The seizure frequencies from visit 2–5 were compared with that at visit 1. The results showed that the decrease in the seizure frequency in every 4-week period during the maintenance period was statistically significant (*P* < 0.001; Table 2).

**Table 2:** Paired *t*-test for seizure frequency in maintenance subjects vs. baseline (visit 1).

	**Mean**	**SD**	**95% CI lower upper**		***t***	***P***
			**lower**	**upper**		
Visit 2	7.25	12.24	4.88	9.62	6.07	<0.001
Visit 3	7.68	13.46	5.06	10.3	5.82	<0.001
Visit 4	8.88	13.82	6.17	11.6	6.49	<0.001
Visit 5	9.33	12.7	6.74	11.91	7.16	<0.001

SD, standard deviation; CI, confidence interval.

It should be noted that the ≥50% response rate was 76.19% at visit 2, 81.73% at visit 3, 94.12% at visit 4, and 95.79% at visit 5, compared with that at visit 1, respectively.

### Secondary outcomes

#### Seizure-free rate

The statistical analyses of the data of the seizures of the subjects from visits 2 to 5 showed that 40 (40/105, 38.10%) subjects at visit 2 had no seizures since the last visit. At visit 3, 53 cases (53/104, 50.96%) were seizure-free, whereas there were 66 cases (66/102, 64.71%) at visit 4, and 67 (67/95, 70.53%) at visit 5. That is, the seizure-free rates from visits 2 to 5 were 38.10, 50.96, 64.71, and 70.53%, respectively. From visits 2 to 5, a total number of 34 subjects (34/107, 31.78%) had no seizures, and the seizure-free rate during the whole maintenance period was 31.78% (Table 3).

**Table 3:** Seizure-free rate for visits 2–5 compared to visit 1.

	**Seizure (%)**	**Seizure free (%)**	**χ^2^**	***P***
Visit 2	65 (61.90)	40 (38.10)	47.78	<0.001
Visit 3	51 (49.04)	53 (50.96)	70.1	<0.001
Visit 4	36 (35.29)	66(64.71)	98.22	<0.001
Visit 5	28 (29.47)	67 (70.53)	109.76	<0.001

#### Retention rate

From visits 2 to 5, 105 cases (105/107, 98.13%) were using the test drug at visit 2, 104 cases (104/107, 97.19%) at visit 3, 102 cases (102/107, 95.33%) at visit 4, and 95 cases (95/107, 88.79%) at visit 5; that is, the retention rates from visits 2 to 5 were 98.13, 97.19, 95.33, and 88.79%, respectively.

#### Follow-up dose

The descriptive analysis of the subjects' maintenance doses for each visiting period showed that the mean doses from visit 1 to 5 were 98.13 ± 23.72 mg/day, 212.38 ± 56.24 mg/day, 222.33 ± 65.20 mg/day, 224.51 ± 66.28 mg/day, and 216.84 ± 59.08 mg/day, respectively.

#### Anxiety and depression evaluation

GAD outcomes in the 107 subjects were as follows: 17 cases scored within 5–9 points, which indicated a mild anxiety disorder; five cases scored within the range of 10–13 points, which indicated a moderate anxiety disorder; and the score of one case was within 14–18 points, which indicated moderate to severe anxiety of the posttreatment GAD outcomes were the following: three out of 94 subjects scored within 5–9 points (mild anxiety disorder), and the rest of the subjects, whose scores were lower than 5 points, were without an anxiety disorder. The mean GAD outcome score before the treatment was 3.00 with a standard deviation of 3.25; after the treatment, the mean GAD score was 1.55 with a standard deviation of 1.66.

The mean NDDI outcome score of the 107 subjects before the treatment was 8.05 with a standard deviation of 2.02, and the mean NDDI outcome score of the 94 remaining subjects after the treatment was 7.01 with a standard deviation of 1.18. A total number of seven subjects (7/107, 6.54%) were defined as depressed before the treatment based on a score threshold of 12, all of whom were depression-free after the treatment. The differences between the pre- and posttreatment values of the GAD and NDDI scores of the 97 subjects were statistically significant, showing better posttreatment outcomes (*P* < 0.001; Table 4).

**Table 4:** GAD/NDDI scores before and after treatment.

**Scores/visit**	**Mean**	***n***	**SD**	***t***	***P***
GAD/visit 1	3	107	3.25	4.78	<0.001
GAD/visit5	1.55	94	1.66		
NDDI/visit1	8.05	107	2.02	5.42	<0.001
NDDI/visit5	7.01	94	1.18		

SD, standard deviation; GAD, generalized anxiety disorder-7; NDDI, neurological disorders depression inventory.

### Safety analysis

#### Adverse events

In the study, one subject experienced an adverse event during the trial, which was dizziness, which was of grade 1 in severity, paroxysmal, and occurred three times. The onset time was on the 122nd day of the trial. The adverse event was considered possibly related to the treatment with LCM. However, the dose was not associated with this effect and thus remained unchanged. The adverse event improved during the follow-up period. During the trial, no subjects experienced serious adverse events.

## Discussion

In recent years, LCM has been widely applied in drug-resistant patients with epilepsy. The present multicenter, real-world observational study is the first investigation assessing the efficacy and safety of nationally produced generic LCM as the first add-on treatment in Chinese adults with focal epilepsy. This domestic generic LCM is bioequivalent to the original drug in Chinese healthy male and female subjects under fasting conditions (19). China has a large population, and nearly 10 million patients with epilepsy are estimated to be living in China (20). However, only approximately one-third of them are well-controlled, whereas the remaining patients receive either inadequate or inappropriate treatment (21). Subjects with focal-onset seizures and a failure of the first antiepileptic drug treatment were enrolled. The results of this current non-interventional study, which is more in accordance with real-world clinical practice, enrolling a cohort of adults with focal epilepsy suggest that LCM is effective and generally well-tolerated as a first add-on treatment for uncontrolled seizures.

It has been reported that after the failure of the first ASM in adult patients with epilepsy, combination therapy may increase the probability of seizure freedom than the additional dose or the alteration of the initial drug previously administered (7). We collected and considered a number of different viewpoints when selecting the first add-on therapy drug due to the emergence of a broad spectrum of new-generation anti-seizure medication (22, 23). The number of prospective studies of the comparative effectiveness of newer-generation ASM as an add-on therapy in drug-resistant epilepsy is increasing, but most of them are used for the treatment of focal epilepsy. Meanwhile, adverse events should be noticed when combination therapy has been started. In the past decade, the most common clinically used drugs as initial or add-on therapy have probably been carbamazepine or oxcarbazepine (24). However, their administration in Chinese patients should be intensively monitored due to the occurrence of severe dermatological adverse reactions and even Stevens–Johnson syndrome and toxic epidermal necrolysis (25–27).

In terms of efficacy, in the current study, the response rate of ≥50% reduction of seizure frequency at visit 2 was 76.19%, which was 81.73% at visit 3, 94.12% at visit 4, and 95.79% at visit 5. The seizure-free rates from visits 2 to 5 were 38.10, 50.96, 64.71, and 70.53%, respectively. From visits 2 to 5, a total number of 34 subjects (34/107, 31.78%) had no seizures. One major limitation of the current study is the rather short-term follow-up period (24 weeks) for the evaluation of the response rate and seizure-free rate, resulting in an inadequate observation and documentation of the sustained response that is clinically meaningful. An ILAE consensus (28) states that at least 1 year of observation time is required to define the absolute seizure-free rate in drug-resistant epilepsy as it is the only measurement associated with significantly improved quality of life. Nonetheless, it also has been reported that LCM add-on treatment may not exert beneficial effects in patients after its administration for longer than 3 months (29, 30). Therefore, the response rate within 3 months usually predicts longer-term efficacy (31–33). The results obtained in the present study in patients with epilepsy with initial monotherapy suggest that a significant decrease in seizure frequency and even seizure freedom may be achieved during the follow-up duration by the administration of LCM as a first add-on antiepileptic drug.

The response rates are better than in previous studies, in which LCM was added to more than one ASM and in the later course of epilepsy. In previous similar studies, one of these is a study evaluating the efficacy of LCM as the first or later add-on therapy. The ≥50% response rate achieved was 70.3%, and the seizure-freedom rate for the 6-month follow-up period was 26.5% in patients with focal epilepsy and 70% responding within seizure frequency (34). Another study used LCM as an add-on therapy based on the concomitant application of one to three ASM. Overall, in the last 3 months of the 6-month follow-up duration, seizure freedom was achieved in 45.5% of the patients, and the ≥50% response rate was 72.5%. The application of LCM as the first add-on therapy resulted in a seizure-free rate of 60.5% and a ≥50% response of 82.1% (33). Therefore, lower rates of seizure freedom and ≥50% responses were observed in patients receiving more than one ASM before the addition of LCM. It is understandable that seizure freedom was more commonly achieved in patients treated for seizure when added as a first than second or later add-on.

The antiepileptic efficacy of LCM was accompanied by improvements in the quality of life and in patient- and physician-rated overall clinical health status. In the present study, emotions and mood were assessed using the GAD and NDDI scales (35, 36), whose posttreatment scores were significantly better than the pretreatment ones. It is noteworthy that only one subject experienced an adverse event during the trial, which was mild dizziness and paroxysmal, which occurred three times. We did not find any other types of AEs in our patients. However, due to the observational nature of this investigation, the small sample size of the enrolled subjects, and the short-term follow-up duration, the interpretation of comorbidity should be accepted with caution. We will perform subgroup analysis and focus on the combination strategy in further study with a larger sample of patients with epilepsy.

## Conclusion

Choosing the first add-on therapy is of critical importance in cases that are non-responsive to the first ASM. Specifically, patients taking one ASM continually in the past 4 weeks and having had at least four episodes every 28 days during the 8-week retrospective baseline period were included. They received oral LCM administration as add-on therapy and were subjected to a further follow-up period of 24 weeks in each center in China. In the present multicenter real-world cohort study, the effect and safety assessment of domestically produced generic LCM in China as the first add-on therapy in adult focal epilepsy was positive. The comorbidities, anxiety, and depression scores of the subjects were significantly improved after treatment compared with the baseline. Nevertheless, further prospective studies with a long-term follow-up period are needed to confirm the findings.

## Data availability statement

The raw data supporting the conclusions of this article will be made available by the authors, without undue reservation.

## Ethics statement

Written informed consent was obtained from the individual(s) for the publication of any potentially identifiable images or data included in this article.

## Author contributions

XW and DZ were involved in the study conception and design. WLiu, WLi, PW, HZ, and EZ performed data analysis and interpretation. WLiu and XW were involved in drafting the article. All authors have provided approval of the final submitted version of the manuscript.
